# RNF183 promotes proliferation and metastasis of colorectal cancer cells via activation of NF-*κ*B-IL-8 axis

**DOI:** 10.1038/cddis.2017.400

**Published:** 2017-08-10

**Authors:** Rong Geng, Xin Tan, Jiangxue Wu, Zhizhong Pan, Min Yi, Wei Shi, Ranyi Liu, Chen Yao, Gaoyuan Wang, Jiaxin Lin, Lin Qiu, Wenlin Huang, Shuai Chen

**Affiliations:** 1 Sun Yat-sen University Cancer Center, State Key Laboratory of Oncology in South China, Collaborative Innovation Center of Cancer Medicine, Guangzhou, P.R. China; 2 School of Food Science and Engineering, Yangzhou University, Yangzhou, P.R. China; 3 Guangdong Provincial Key Laboratory of Tumor Targeted Drugs and Guangzhou Enterprise Key Laboratory of Gene Medicine, Guangzhou Doublle Bioproducts Co. Ltd., Guangzhou, P.R. China; 4 Guangdong Esophageal Cancer Institute, Sun Yat-sen University Cancer Center, Guangzhou, P.R. China

## Abstract

Colorectal cancer (CRC) is one of the most common malignant tumors worldwide, which is a heterogeneous disease and main risk factors are associated with inflammation, family history, genetic mutations, epigenetics, and so on. Ring finger domain proteins have been reported involved in carcinogenesis, whereas their roles in CRC are rarely studied. Here, we reanalyzed the expression of 202 RNF family members in CRC using published microarray data from GEO database and found that RNF183 is markedly upregulated in tumor tissues. RNF183 high expression is significantly associated with tumor size (*P*=0.012), tumor invasive depth (*P*=0.004), TNM stage (*P*=0.01), and distant metastasis (*P*=0.009). CRC patients with high expression of RNF183 have poor overall survival (*P*<0.001) and progression-free survival (*P*<0.001). Functional studies suggest that RNF183 facilitates growth, migration, and invasion of CRC cells *in vitro* and promotes tumor proliferation and metastasis *in vivo*. Mechanistically, RNF183 activates NF-*κ*B signal pathway through P65 and stimulates the transcription of multifunctional chemokine IL-8. Blockage of NF-*κ*B by small molecule inhibitor or depletion of IL-8 by siRNA attenuates the function of RNF183 to promote cell migration. Moreover, the regulation of RNF183 on IL-8 transcription and cell viability/motility is dependent on its E3 ubiquitin ligase activity. Our study provided proof of principle to show that RNF183 promotes proliferation and metastasis of CRC cells via activation of NF-*κ*B-IL-8 axis.

Colorectal cancer (CRC) is the third most common malignancy of cancer morbidity worldwide.^1^ The overall neoplastic lethality rate of CRC is ~50%.^1^ The mechanisms underlying CRC is complex and many key pathways and genes are involved, including *APC*, *Wnt*, *β-catenin*, *EGFR/RAS/RAF/MEK*, *TFG-β*, and so on.^2, 3^ Although targeted drugs such as anti-EGFR monoclonal antibodies cetuximab and panitumumab have been use for the treatment of metastatic colorectal cancer (mCRC), <20% of mCRC patients with wild-type *KRAS* benefit from these drugs when they were used as monotherapy.^4, 5^ On the other hand, targeting downstream kinases of EGFR, such as BRAF and MEK, shows less efficacy in CRC therapy.^4, 6^ Thus, it is critical to discover new mechanisms that drive the initiation and development of CRC to provide new targets for therapy.

The RING finger (RNF) protein family is a complex set of proteins containing a RNF domain, which typically includes 40–60 amino acids.^7^ More than 200 RNF family genes have been identified, and many of which exhibit wide range of functions in different biological and pathological processes.^8^ Many RNF family members have been reported to play key roles in carcinogenesis and development. For instance, RNF31 is a subunit of linear ubiquitin chain assembly complex (LUBAC) and is affected by germline polymorphisms among activated B-cell-like subtype of diffuse large B-cell lymphoma patients (~7.8%), which increase LUBAC enzymatic activity thus in turn activate NF-*κ*B and promote tumorigenesis.^9^ High-frequent mutation of RNF43 is observed in gastrointestinal cancer including CRCs and cystic pancreatic tumors, which sensitize tumor cells to Wnt inhibitors.^10, 11^ Identification of more RNF family members associated with cancer, including colorectal carcinoma, will help to understand the process of carcinogenesis and to develop new therapeutic strategies.

In the current study, we reanalyzed GEO data set GSE8671 and compared the expression of 202 RNF family genes between colorectal adenoma and adjacent normal tissues. Our analyses revealed significant increased expression of *RNF183* in tumor tissues. Although RNF183 is found upregulated in inflammatory bowel disease (IBD), a disease highly associated with CRC, and activates classic NF-*κ*B pathway,^12, 13, 14^ its role in cancer cells remains largely unknown. Here, we report that RNF183 is an independent prognostic factor in CRC patients, and high RNF183 level is closely related to tumor size, invasive depth, TNM stage and distant metastasis. RNF183 upregulates the expression of multifunction chemokine IL-8 through NF-*κ*B pathway and promotes cancer cell growth, migration, invasion and metastasis *in vitro* and *in vivo*. Therefore, our study indicates that RNF183 is a CRC associated gene and could serve as a target for the development of new therapeutic strategies.

## Results

### RNF183 is upregulated in CRC

**Figure 1:**
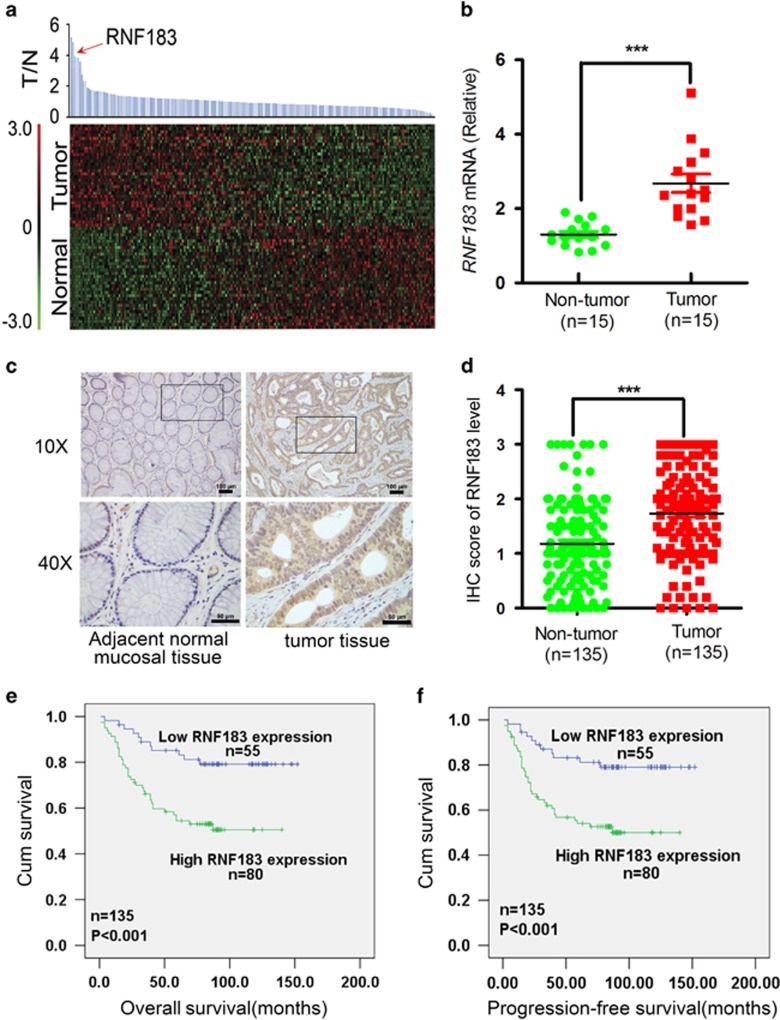
The expression of RNF183 is elevated in human colorectal cancer tissues. (**a**) Reanalyzing the expression of RNF family members between normal and tumor tissues in CRC from GEO data set GSE8671. (**b**) The relative mRNA expression of *RNF183* was evaluated by RT-PCR in 15 paired human normal colorectal tissues and CRC tissues. (**c**) IHC staining of RNF183 expression in CRC tissues and adjacent normal mucosal tissues. Scale bar: 100 *μ*m (× 10) or 50 *μ*m × (40). (**d**) IHC analyses of RNF183 in paired CRC cancerous and noncancerous tissues from 135 patients. (**e** and **f**) Kaplan–Meier survival analysis of the association between RNF183 expression and overall survival (**e**) or progression-free survival (**f**) in 135 patients. *P*-value was determined by long rank test. ***P<0.001

To identify RNF members that are aberrantly expressed in CRC, we compared gene expression of 202 RNF genes between cancerous and normal adjacent tissues using GEO data set GSE8671. The analysis showed that 70 RNF family members are elevated in CRC tissues (Figure 1a). The top two genes among these upregulated RNF members, *RNF206/TRAIP* and *RNF90/TRIM7* have been reported to drive tumor formation.^15, 16^ *RNF183* ranks the third in all elevated genes with unknown function in CRC, and was chosen for further analysis. Quantitative RT-PCR experiment in 15 paired CRC and corresponding normal tissues confirmed elevated *RNF183* mRNA level in cancerous tissues (Figure 1b). Furthermore, we detected the level of RNF183 protein in 135 paired CRC tissues using immunohistochemistry (IHC), and the staining was scored for each sample (four intensity levels: 0–3; Supplementary Figure 1). The expression of RNF183 is significantly higher in tumor tissues than in adjacent non-tumor tissues (Figures 1c and d). High and low RNF183 staining was observed in 80 (59.3%) and 55 (40.7%) CRC tissue samples, respectively (Table 1). RNF183 high expression was significantly associated with tumor size (*P*=0.012), tumor invasive depth (*P*=0.004), TNM stage (*P*=0.01), and distant metastasis (*P*=0.09) (Table 1). Kaplan–Meier survival analysis showed that patients with high RNF183 expression had shorter overall survival (OS, Figure 1e) and progress-free survival (PFS, Figure 1f). Multivariate Cox proportional hazards regression analysis showed that RNF183 level is an independent prognostic factor for PFS (*P*<0.05, hazard's ratio (HR)=0.384, 95% CI=0.172–0.861) and OS (*P*<0.05, HR=0.364, 95% CI=0.159–0.831) (Table 2).

### RNF183 accelerates the growth of CRC cells

**Figure 2:**
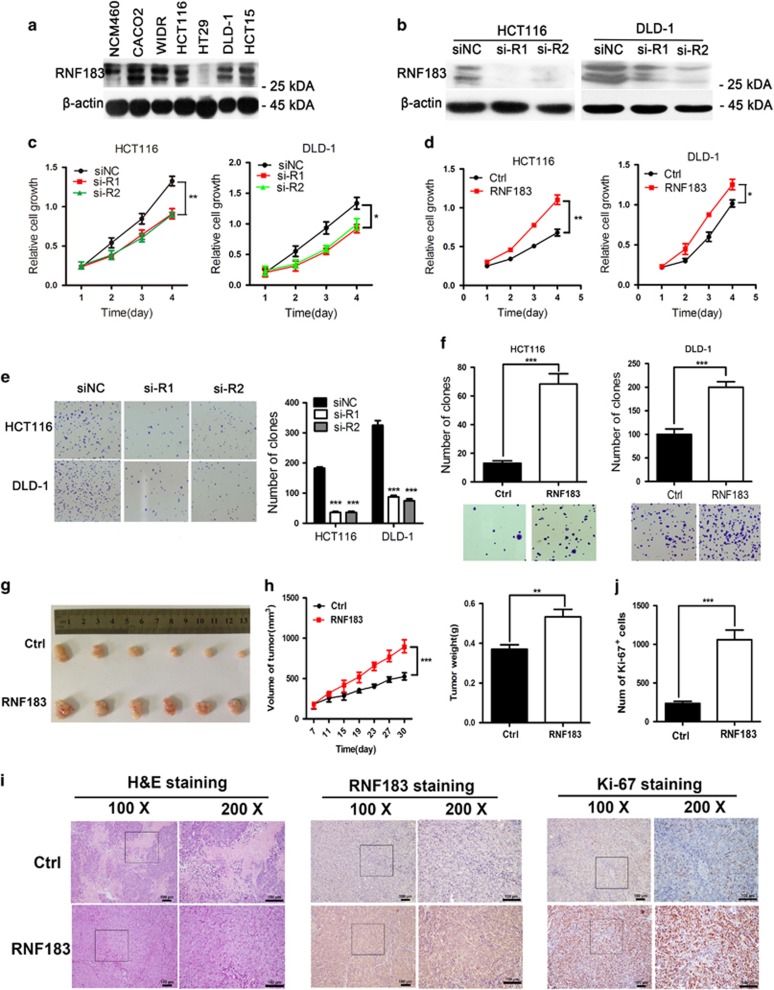
RNF183 promotes proliferation of CRC cells *in vitro* and *in vivo*. (**a**) Western blots detect endogenous RNF183 protein expression in human normal colorectal epithelial cell line and CRC cells. (**b**) RNF183 knockdown efficiency in two CRC cell lines was levels were examined by western blots. (**c** and **d**) Effects of RNF183 silencing (**c**) or overexpression (**d**) on proliferation of HCT116 and DLD-1 cells was monitored by MTT assays. Mean±S.D. (*n*=6). (**e**and **f**) Effects of RNF183 silencing (**e**) or enforced expression (**f**) on the colony formation of HCT116 and DLD-1 cells. (**g**–**j**) RNF183 overexpression accelerates tumor growth *in vivo*. (**g**) Tumor pictures from mice inoculated with stable RNF183 overexpression cell line DLD-1 or control. (**h**) Growth curve of tumor volume measured on indicated days (left) and tumor weight at the end of experiment (right). Mean±S.D. (*n*=6). (**i**) Photographs exhibited the H&E staining (left), IHC staining for RNF183 (middle) and ki-67 (right) in tumors. Scale bar: 100 *μ*m. (**j**) Number of Ki-67 positive cells in control and RNF183 stable expression tumors. *P<0.05, **P<0.01, ***P<0.001

As unlimited growth, invasiveness, and metastasis are hallmarks of malignancy,^17^ we tried to explore the role of RNF183 in progression of CRC. The endogenous RNF183 expression is higher in CRC cells than in NCM460, a normal colorectal epithelial cell (Figure 2a). We silenced RNF183 expression in DLD-1 and HCT116 cells, and the knockdown efficiency is shown in Figure 2b. RNF183 knockdown suppressed the growth of DLD-1 and HCT116 cells (Figure 2c), whereas RNF183 overexpression accelerates the proliferation of both cell lines in MTT assays (Figure 2d). Furthermore, colony formation assay shows that RNF183 silencing significantly reduced size and number of colonies (Figure 2e), and its enforced expression shows opposite phenomenon (Figure 2f). These results indicated that RNF183 promotes cell growth *in vitro*.

We next investigated whether RNF183 affects the proliferation of CRC cells *in vivo*. Control and RNF183 stable overexpression DLD-1 cells were subcutaneously injected into BALB/c nude mice to generate xenograft tumors. The results showed that RNF183 enforced expression significantly promote tumor growth (Figure 2g). Tumors with RNF183 overexpression have larger size and higher weight than control tumors (*P*<0.01) (Figure 2h). RNF183 overexpression was confirmed by IHC staining (Figure 2i), and tumors with RNF183 overexpression have higher percentage of Ki-67-positive cells than control tumors (Figures 2i and j).

### RNF183 promotes migration, invasion, and metastasis of CRC cells

**Figure 3:**
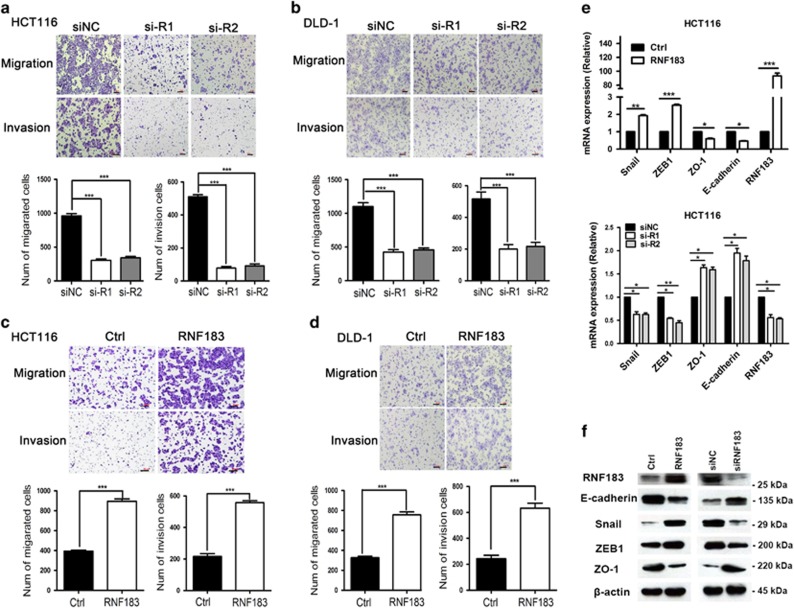
RNF183 prmotes migration, invasion and epithelial–mesenchymal transition (EMT) of CRC cells. (**a** and **b**) Effects of RNF183 silencing on migration and invasion of HCT116 (**a**) and DLD-1 (**b**) cells evaluated by transwell assays. Mean±S.D. (*n*=3). (**c**and**d**) Effects of RNF183 overexpression on migration and invasion of HCT116 (**c**) and DLD-1 (**d**) cells detected by transwell assays. Mean±S.D. (*n*=3). (**e**) Effects of RNF183 overexpression (upper) and silencing (bottom) on the mRNA abundance of selected EMT markers in HCT116 cells. (**f**) Effects of RNF183 overexpression (left) and silencing (right) on the protein expression of selected EMT markers in HCT116 cells. Scale bar: 100 *μ*m. *P<0.05, **P<0.01, ***P<0.001

Subsequently, we explored the function of RNF183 on the motility of CRC cells. In transwell assays, RNF183 knockdown significantly impaired the migration and invasion ability of HCT116 (Figure 3a) and DLD-1 (Figure 3b) cells, whereas RNF183 overexpression largely facilitated the motility of both cell lines (Figures 3c and d). Given that epithelial–mesenchymal transition (EMT) is a critical event involved in the initiation of tumor invasion, the impact of RNF183 on the expression of several EMT markers was analyzed in HCT116 cells. RNF183 overexpression significantly induced the transcription of *Snail* and *ZEB1* and repressed the mRNA abundance of *ZO-1* and *E-cadherin* (Figure 3e, upper). RNF183 knockdown, however, repressed *Snail* and *ZEB1* expression but induced *ZO-1* and *E-cadherin* transcription (Figure 3e, bottom). The regulation of these EMT markers was further confirmed by western blots (Figure 3f).

**Figure 4:**
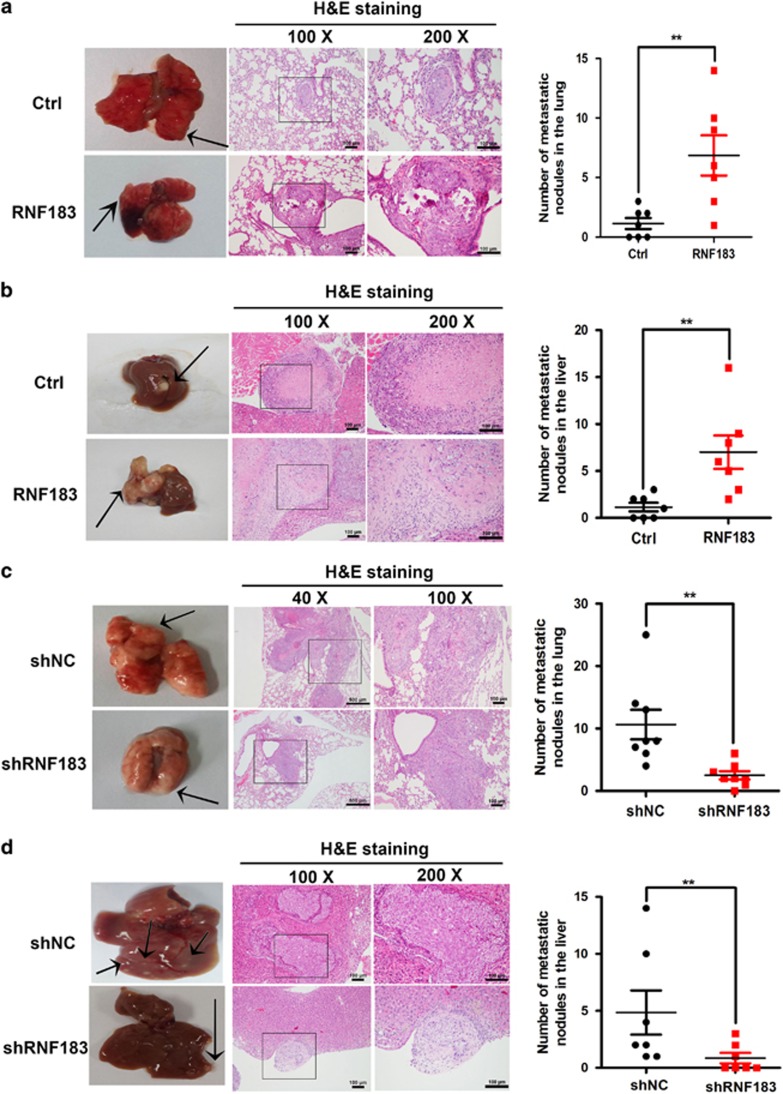
RNF183 promotes metastasis of CRC cells *in vivo*. (**a**) Lungs were removed from mice injected with control or DLD-1 stable cell line with RNF183 overexpression. Pulmonary histopathology was examined by H&E staining. Mean±S.D. (*n*=7). (**b**) Livers were removed from mice injected with control or DLD-1 stable cell line with RNF183 overexpression. Hepatic histopathology was examined by H&E staining. Mean±S.D. (*n*=7). (**c**) Lungs were dissected from mice injected with control or RNF183 stable silenced HCT116 cells. Pulmonary histopathology was examined by H&E staining mean±S.D. (*n*=8). (**d**) Livers were dissected from mice injected with control or RNF183 stable silenced HCT116 cells. Hepatic histopathology was examined by H&E staining. Mean±S.D. (*n*=7). Scale bar: 100 *μ*m (× 100 and × 200) or 500 *μ*m (× 40). **P<0.01

The above *in vitro* results encouraged us to evaluate the role of RNF183 on tumor metastasis *in vivo*. We developed lung and hepatic metastasis models by injecting control or RNF183 stable overexpression DLD-1 cells into the lateral tail veins or spleens of nude mice for 8–10 weeks. Compared with control groups, RNF183 enforced expression increased the number of metastatic pulmonary nodules (Figure 4a) and liver nodules (Figure 4b). In knockdown experiments, RNF183 silencing reduced lung (Figure 4c) and hepatic (Figure 4d) metastasis of HCT116 cells. These data suggested that RNF183 promotes migration, invasion and metastasis of CRC cells *in vitro* and *in vivo*.

### RNF183 regulates *IL-8* transcription through NF-*κ*B signaling

**Figure 5:**
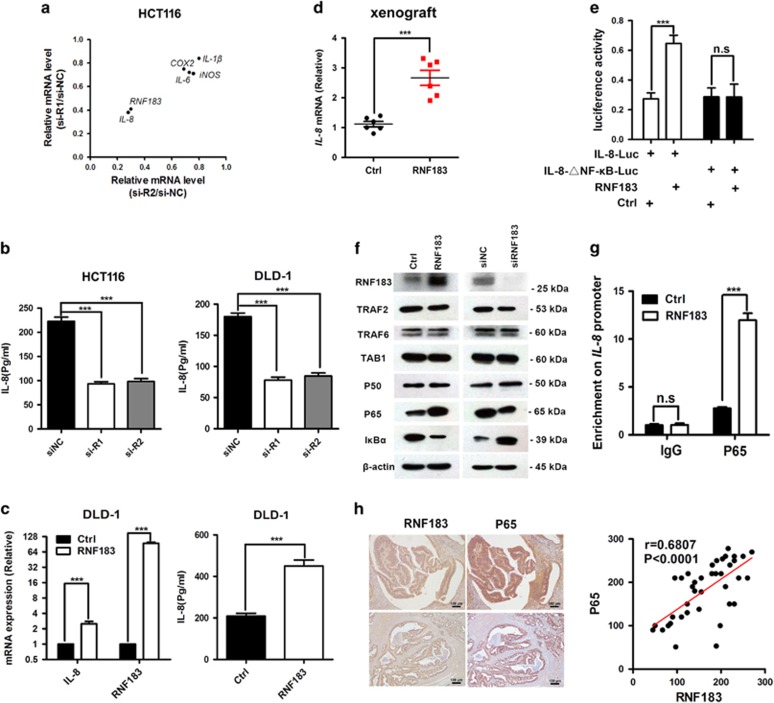
RNF183 promotes *IL-8* transcription through NF-*κ*B. (**a**) The effects of RNF183 knockdown on the mRNA abundance of several NF-*κ*B downstream genes in HCT116 cells. (**b**) Knockdown RNF183 expression significantly reduced IL-8 secretion in HCT116 (left) and DLD-1 (right) cells. (**c**) Enforced RNF183 expression augments *IL-8* transcription (left) and IL-8 secretion (right) in DLD-1 cells. (**d**) Stable RNF183 overexpression increased *IL-8* transcription in xenograft tumors as shown in Figure 2g. (**e**) Effects of RNF183 on the activity of luciferase reporter with wild-type or NF-*κ*B binding site deleted (△NF-*κ*B) *IL-8* promoter in HCT116 cells. (**f**) Expression of several proteins in NF-*κ*B pathway was examined by western blots with enforced RNF183 expression in HCT116 cells or with RNF183 knockdown in DLD-1 cells. (**g**) Chromatin immunoprecipitation (ChIP) assays were carried out to determine the binding of P65 on *IL-8* promoter with or without RNF183 enforced expression. (**h**) The expression of P65 and RNF183 were evaluated in forty CRC tissues. The correlation of these two proteins and the significance were also calculated. Mean±S.D. (*n*=3). Scale bar: 100 *μ*m. ***P<0.001

The function of RNF183 in growth and metastasis of CRC cells predicts its role in regulating oncogenic pathways. It had been reported that RNF183 activates NF-*κ*B in IBD,^12^ so we performed qPCR to evaluate the influence of RNF183 on several NF-*κ*B downstream targets. RNF183 knockdown repressed the transcription of *IL-8*, *IL-6*, *COX2*, *iNOS* as well as *IL-1β*, and the reduction of *IL-8* is the most significant among these genes (Figure 5a). ELISA assays confirmed that RNF183 knockdown markedly decreased the level of secreted IL-8 in HT116 and DLD-1 cells (Figure 5b). On the contrary, RNF183 enforced expression significantly induced IL-8 level at both mRNA and protein level in DLD-1 cells (Figure 5c). Elevated *IL-8* transcription was also observed in DLD-1 xenograft tumors with RNF183 overexpression (Figures 2g and 5d).

In order to elucidate whether NF-*κ*B is required for RNF183 to induce *IL-8* transcription, we performed luciferase assays using wild-type *IL-8* promoter (IL-8-Luc) or *IL-8* promoter with NF-*κ*B binding site deletion (IL-8-△NF-*κ*B-Luc). As shown in Figure 5e, RNF183 activates IL-8-Luc but not IL-8-△NF-*κ*B-Luc, suggesting that NF-*κ*B is indispensable for RNF183 to regulate *IL-8* transcription. Furthermore, we assessed the effect of RNF183 on the expression of several key proteins in NF-*κ*B pathway. Among all tested proteins, P65 was significantly suppressed or evaluated according to RNF183 knockdown or overexpression (Figure 5f), respectively. As P65 is a core transcriptional factor drives *IL-8* expression, we detected the enrichment of this protein on *IL-8* promoter. Chromatin immunoprecipitation (ChIP) assays were performed and increased binding of P65 on *IL-8* promoter was found upon RNF183 enforced expression (Figure 5g). Finally, IHC staining showed a positive correlation of RNF183 and P65 protein level in 40 CRC tissue sections (Figure 5h). Taken together, these data suggest that RNF183 induced P65 expression and subsequent NF-*κ*B activation is responsible to stimulate *IL-8* transcription in CRC cell, xenograft and pathological tissues.

### The oncogenic function of RNF183 is dependent on NF-*κ*B-IL-8 axis

**Figure 6:**
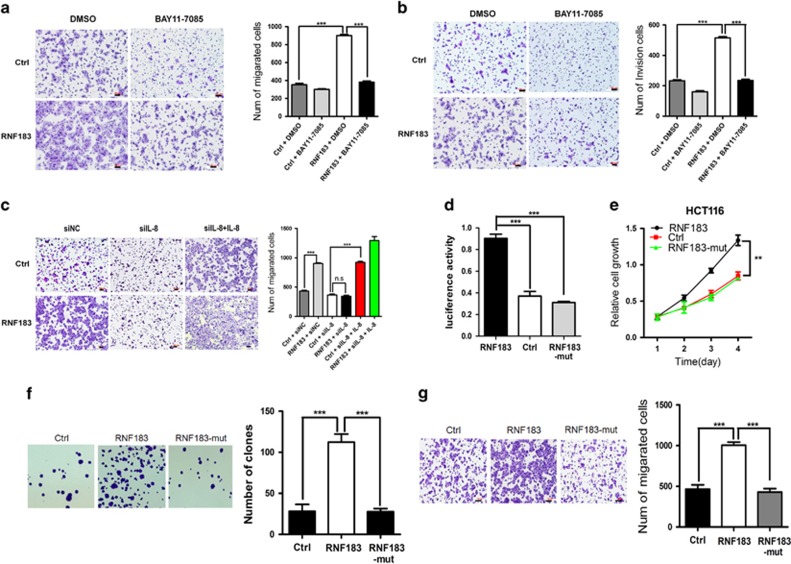
NF-*κ*B-IL-8 axis is indispensible for the oncogenic function of RNF183. (**a**and **b**) NF-*κ*B inhibitor BAY11-7085 attenuates RNF183 overexpression induced migration (**a**) and invasion (**b**) of DLD-1 cells. (**c**) HCT116 cells were co-transfected with RNF183 plasmid and siRNA-targeting IL-8 as indicated. Recombinant IL-8 was also supplemented in the knockdown group to rescue the phenotype. The pictures of migrated cells (left) and quantification (right) were showed. (**d**) Luciferase activity of *IL-8* promoter was evaluated in HCT116 cells with overexpression of control plasmid, wild-type RNF183 and truncated RNF183 without E3 ubiquitin ligase activity. (**e–g**) Effects of wild-type and truncated RNF183 on proliferation (**e**), colony formation (**f**) and migration (**g**) of HCT116 cells. Mean±S.D. (*n*=3). Scale bar: 50 *μ*m. **P<0.01, ***P<0.001

Encouraged by the above results, we decided to challenge whether targeting NF-*κ*B-IL-8 axis could attenuate the oncogenic function of RNF183. We treated DLD-1 cells with NF-*κ*B inhibitor BAY11-7085, and found that this compound could inhibit RNF183 overexpression induced cell migration (Figure 6a) and invasion (Figure 6b). In another assay, transfection of IL-8 targeted siRNA inhibits RNF183 induced migration of DLD-1 cells (Figure 6c), and reduced IL-8 secretion is confirmed by ELISA assay (Supplementary Figure 2). As expected, this phenomenon was rescued by adding recombinant IL-8 protein to the culture medium of knockdown cells (Figure 6c), suggesting an oncogenic role of IL-8 as previously reported.^18, 19^ Next, we generated a truncated form of RNF183 (RNF183-mut) without E3 ubiquitin ligase activity by deleting its RING domain (amino acids 1–59), and compared its function with wild-type RNF183. This truncation largely abolished the function of RNF183 in activating *IL-8* promoter (Figure 6d) and promoting the growth (Figure 6e), colony formation (Figure 6f), and migration (Figure 6g) of HCT116 cells. These results thus suggest that the oncogenic function of RNF183 is largely dependent on its E3 ubiquitin ligase activity to regulate NF-*κ*B-IL-8 axis.

## Discussion

Overwhelming evidences have shown that inflammation has crucial roles in tumor development, including initiation promotion, malignant conversion, invasion, and metastasis.^20^ Studies even show that inflammatory responses can eliminate the effects of cancer therapy.^21^ A classic inflammation-driven cancer is a subtype of CRC named colitis-associated colon cancer (CAC), which is usually developed from IBD.^14, 22^ Other subtypes of colorectal tumor also demonstrate vigorous inflammation and aberrant high level of inflammatory cytokines.^23, 24^ Moreover, a large proportion of CRC tumors and CRC cell lines exhibits increased activity of NF-*κ*B signal pathway.^25^ However, the underlying molecular mechanism that drive the hyperactivation of inflammatory responses in CRC remains largely unknown.

Previous study indicated that RNF183 is upregulated in intestinal epithelial cells of IBD patients and TNBS-induced colitis mice.^12^ In our study, we provided evidences to show that RNF183 is aberrantly overexpressed in CRC cell lines and tissues. Patients with high expression of RNF183 have shorter OS and PFS, indicating that RNF183 could serve as an independent prognostic factor in CRC. Enforced expression of RNF183 promotes proliferation, invasion, and metastasis of tumor cells, whereas opposite phenomenon were observed in RNF183 knockdown experiments, indicating that RNF183 functions as an oncogene in the pathological process of CRC. Elevated expression of RNF183 in both IBD and CRC suggested a possibility that RNF183 may contribute to the transformation from inflammation to malignancy. In IBD, RNF183 activates NF-*κ*B pathway through ubiquitination and degradation of I*κ*B*α*;^12^ In CRC cells, we found that RNF183 induced IL-8 expression in an E3 ubiquitin ligase-dependent manner. IL-8 is transcriptional controlled by transcription factors NF-*κ*B and AP-1,^18^ and we confirmed that RNF183 increased the abundance of NF-*κ*B transcription factor P65 and its enrichment to *IL-8* promoter in CRC cells. In accordance with previous reports that NF-*κ*B signal pathway and its downstream chemokine IL-8 contributes to inflammation and to every step of CRC progression proliferation, migration and invasion,^26, 27, 28^ we found that NF-*κ*B inhibition and IL-8 depletion eliminates the tumor-promoting activity of RNF183.

Taken together, our data indicate that RNF183 contributes to the transformation from inflammation to malignancy in CRC, and predicts the application of NF-*κ*B inhibitors or IL-8 antagonists for precision therapy of CRC with elevated RNF183 expression.

## Materials and methods

### Bioinformatics analyses

The expression of RNF family genes were downloaded from GEO database (https://www.ncbi.nlm.nih.gov/geo/) under the accession number GSE8671. The significance of gene expression between normal and tumor tissues were calculated using Excel 2007. The heatmap was drawn using Genesis software (http://genome.tugraz.at/genesisclient/genesisclient_description.shtml) For genes with multiple probe-sets, the one with lowest *P*-value between normal and tumor tissues were chosen for further analyses.

### Plasmids, antibodies, and reagents

The plasmid of pcDNA4-myc/his-RNF183 was constructed by the company of genewiz (Suzhou, China). A truncated form of RNF183 without amino acids 1–59 was generated by PCR and subsequent molecular cloning into pcDNA4-myc/his vector. IL-8 and IL-8-△NF-*κ*B reported plasmids were kindly provided by Professor Hongbin Shu (Wuhan University, China). The antibodies used were listed as follows: human anti-RNF183 antibody (1:1000 for western blot, 1:200 for IHC, ab197321, Abcam, Cambridge, UK), anti-TAB1 antibody (1;1000, #3226 CST), anti-TRAF2 antibody (1:200, sc-136999, Santa Cruz Biotech, Santa Cruz, CA, USA), anti-TRAF6 (1:200, sc-7221, Santa Cruz), anti-NF-*κ*B p65 antibody (1:1000 for western blot, 1:200 for IHC, 1:100 for CHIP, #8242, CST), anti-NF-*κ*B P50 antibody (1:1000, #12540, CST, Boston, MA, USA), anti-ki-67 antibody(1:1000, #12202, CST), anti-*β*-actin (1:200, sc-47778, Santa Cruz), anti-mouse IgG HRP conjugate (1:8000, W402B, Promega, Madison, WI, USA), anti-Rabbit IgG HRP conjugate (1:2000, #7074P2, CST), normal rabbit IgG (1 μg for ChIP, sc-3888, Santa Cruz). Recombinant human IL-8 was purchased from Novus (Littleton, CO, USA) (NBP2-34905). The siRNAs (ribobio Company, Beijing, China) sequences were listed in supplementary table 1.

### Cell lines and cell culture

All CRC cell lines were purchased from the American Type Culture Collection (ATCC) and cultured in a humidified atmosphere of 5% CO_2_ at 37 °C. HCT116 and HT29 were cultured in McCoy's 5A Medium (MC5A, Gibco, Carlsbad, CA, USA); NCM460, CACO2, and WIDR were cultured in Dulbecco's Modified Eagle Medium; DLD-1 and HCT15 were cultured in RPMI 1640 medium. A total of 10% fetal bovine serum (FBS) was supplemented in the culture medium. Thawed cells from liquid nitrogen were used for within the first three passages.

### Patient samples

Fifteen CRC specimens and their matched adjacent normal tissue (for *RNF183* mRNA level analysis by RT-PCR) were obtained from Sun Yat-sen university cancer center (Guangzhou, China) with the agreement of the patients. A total of 135 CRC patients who underwent resection of primary CRC between January 1999 and December 2005 at the Sun Yat-sen University Cancer Center were chosen for IHC assays. The detailed selection criterion for CRC patients was described previously.^29^

### Cell viability assay

Cell growth was monitored by MTT assay. Treated or control cells at a density of 3 × 10^3^ per well were grown in the 96-well plates in 0.1 ml full medium at 37 °C for 24 h. Each indicated set had six duplicated wells, and MTT assays were repeated three times according to the manufacturer’s protocol.^29^ Colony formation assays were in triplicate and conducted in six-well flat-bottom plates. The number of visible colonies was counted after staining with 5% crystal violet.

### Migration and invasion assay

Cells were plated into the upper chamber with 8 μm pore (BD Falcon, Franklin Lakes, NJ, USA) at a density of 1 × 10^5^ per well. Chambers without or with Matrigel matrix for cells migration or invasion assays were inserted into matching 24-well plate containing MC5A 20% FBS. Cells in the chamber were cultivated without fetal bovine serum. After 20 h, migrated or invasive cells adhering to the lower surface of chamber were fixed and stained with 5% crystal violet and were counted in five fields of microscope.

### Stable cell lines

For the overexpression experiments, HCT116 and DLD-1 cells were transfected with pcDNA4-myc/his-RNF183 or empty vector pcDNA4-myc/his using Lipofectamine2000 (Invitrogen, Grand Island, NY). After 48 h, the RNF183 stable cells were selected by 300 μg/ml zeocin (Invitrogen) for additional 12 days. The culture medium was renewed every 3 days. For the knockdown experiments, RNF183 and control shRNA lentiviral vector was generated by GenePharma (GenePharma Corporation, Shanghai, China). Lentiviral vectors were transfected into HCT116 and DLD-1 cells prior to 3 μg/ml puromycin selection for 3 days. The stable RNF183 overexpression and knockdown cells were confirmed with qPCR and western blots.

### Luciferase reporter assay

Dual-Luciferase Reporterassays (Promega) were performed according to the manufacturer’s instruction. In brief, HCT116 cells were planted in 24-well plate 24 h prior to the transfection. After another 48 h, Luciferase Assay Reagent II (LAR II) and Stop & Glo Reagent sequentially, and the dual luciferase activity was measured.^30, 31^

### Xenograft tumor growth model

All animal experiments were conducted according to the institutional ethical and safe guidelines (Institutional Animal welfare and Ethics Committee, Sun Yat-sen university cancer center, Guangzhou, China). In total, 5–6-week-old female BALB/c nude mice were used in the experiments (SLAC laboratory animal lnc, Shanghai, China). WT-RNF183 stable overexpressed and Control DLD-1 xenograft model sets were established using 1 × 10^6^ cells injection into the right flanks of subcutaneous tissue of mice. Tumor length and width and mice weight were measured every 2–3 days until the experiment was ended. The tumor volume was calculated based on the conventional formula: V=(L × W^2^)/2, where L and W are the biggest and smallest diameters of the tumor.

### Xenograft tumor metastasis model

For the pulmonary metastasis model, suspensions of stable cell lines were inoculated into the tail vein of nude mice (1 × 10^6^ cells per mouse). For the intrahepatic metastasis model, mice were general anaesthetized by isoflurane, and the spleen was brought outside enterocoelia from 1 cm surgical incision on the left lower abdomen; the stable cell lines were injected into spleen and then, spleen was reset. After 8–10 weeks, the lungs and livers of mice were removed for pathological examination (H&E staining). The number of metastasic nodules in lung and liver were counted under microscope.

### IHC

Protein levels of RNF183 were detected by IHC assay with a peroxidase kit (DAKO, Carpinteria, CA, USA) as described previously.^32^ In brief, after routine deparaffinization, rehydration, and blocking with 0.3% H_2_O_2_ and antigen retrieval, the slides were incubated overnight at 4 °C with anti-RNF183 antibody, followed by incubation with HRP-conjugated secondary antibody and visualized with the EnVision Detection Kit (DAKO). Then, the sections were counterstained with hematoxylin. RNF183 staining intensity and the percentage of corresponding positive area were evaluated by two pathologists who were blinded to clinical parameters. The RNF183 protein levels were presented as H score.^32^

### ELISA and western blots

The secreted IL-8 was monitored by ELISA assays using human IL-8 ELISA kit (Proteintech, Wuhan, China). For western blots, total proteins were extracted from cells using cell lysis buffer containing protease inhibitor (Sigma-Aldrich, St. Louis, MO, USA). For western blots, samples were separated by 6–10% SDS-polyacrylamide gel electrophoresis, followed by transfer to PVDF membrane. After blocking in phosphate-buffered saline containing 5% bovine serum albumin and 0.1% Tween-20, the membrane was incubated with primary antibody at 4 °C overnight, followed by incubation with a peroxidase-linked secondary antibody (CalbioChem, Darmstadt, Germany) at room temperature for 1 h. The signals were detected using Western blotting Luminol Reagent (Santa Cruz Inc.).

### ChIP

ChIP Assay Kit was purchased from Beyotime (Shanghai, China). In brief, 3 × 10^6^ cells with or without RNF183 transfection were cross-linked using 1% formaldehyde for 10 min. DNA fragments were sheared to 200–1000 bp by ultrasound deal on ice. The lysates were slow-whirling overnight at 4 °C with anti-P65 antibody or Rabbit IgG. Then the P65 bound DNA was precipitated and purified, and the enrichment of P65 on *IL-8* promoter was examined by qPCR.

### RNA extraction and RT-PCR

Total RNA was extracted using Trizol (Invitrogen, Carlsbad, CA, USA). The cDNA was synthesiszed from 1 *μ*g of total RNA using M-MLV reverse transcriptase (Promega). SYBR Green was purchased from BIO-RAD (Hercules, CA, USA) for quantitative RT-RCR.^33^ The primer sequence of target genes and reference gene *GAPDH* were listed in Supplementary Table 2.

### Statistical analysis

Statistical analyses were completed using the SPSS version 16.0 (SPSS Inc., Chicago, IL, USA).^34, 35^ Two-tailed *χ*^2^-tests were performed to assess significant associations between RNF183 levels and clinicopathological parameters. Survival analysis was carried out using the Kaplan–Meier method, and the log-rank test was used to compare the survival curves. The Cox proportional hazards model was used to calculate the univariate and multivariate hazards ratios for clinicopathological parameters and the RNF183 level with respect to OS and PFS. Other data were assessed with Student’s two-tailed *t*-tests. *P*<0.05 was considered statistically significant. *P*-value<0.05 was considered statistically significant.

## Figures and Tables

**Table 1:** Clinicopathological characters and their correlation with RNF183 expression

**Variables**	***N* (%)**	**RNF183 low (%)**	**RNF183 high (%)**	***P*-value**
Total case	135	55(40.7%)	80(59.3%)	
				
*Age (years)*				
<65	91 (67.4%)	36 (26.7%)	55 (40.7%)	
⩾65	44 (32.6%)	19 (14.1%)	25 (18.5%)	0.726
				
*Gender*				
Male	76 (56.3%)	29 (21.5%)	47 (34.8%)	
Female	59 (43.7%)	26 (19.3%)	33 (24.4%)	0.492
				
*Tumor location*				
Colon	64 (47.4%)	23 (17%)	41 (30.4%)	
Rectum	71 (52.6%)	32 (23.7%)	39 (28.9%)	0.284
				
*Tumor size(cm)*				
<5	54 (40%)	29 (21.5%)	25 (18.5%)	
⩾5	81 (60%)	26 (19.3%)	55 (40.7%)	0.012^a^
				
*Tumor invasive depth*				
T1–T2	24 (17.8%)	16 (11.8%)	8 (6%)	
T3–T4	111 (82.2%)	39 (28.9%)	72 (53.3%)	0.004^a^
				
*AJCC/TNM stage*				
I–II	63 (46.7%)	33 (24.4%)	30 (22.3%)	
III–IV	72 (53.3%)	22 (16.3%)	50 (37%)	0.01^a^
				
*Lymph node status*				
<1	68 (50.4%)	35 (26%)	33 (24.4%)	
⩾1	67 (49.6%)	20 (14.8%)	47 (34.8%)	0.35
				
*Distant metastasis*				
No metastasis	105 (77.8%)	49 (36.3%)	56 (41.5%)	
Metastasis	30 (22.2%)	6 (4.4%)	24 (17.8%)	0.009^a^
				
*Preoperative CEA (ng/ml)*				
<5	64 (50.4%)	29 (22.8%)	35 (27.6%)	
⩾5	63 (49.6%)	24 (18.9%)	39 (30.7%)	0.414

Abbreviations: CA199, carbohydrate antigen 19–9; CEA, carcino embryonic antigen

The numbers in parentheses indicate the percentages of tumors with a specific clinical or pathologic feature for a given RNF183 subtype

a Statistically significant, *P*<0.05

**Table 2:** Multivariate analysis for PFS and OS in CRC patients

**Variables**	**PFS HR (95% CI)**	***P*-value**	**OS HR (95% CI)**	***P*-value**
Age (year, <65 versus ⩾65)	0.692 (0.355–1.349)	0.28	0.6 (0.34–1.316)	0.244
Gender (male versus female)	0.94 (0.482–1.833)	0.856	0.96 (0.484–1.907)	0.908
Tumor location (colon versus rectum)	0.724 (0.38–1.38)	0.327	0.69 (0.362–1.342)	0.28
Tumor size (cm, <5 versus ⩾5)	0.811 (0.371–1.771)	0.599	0.86 (0.391–1.89)	0.707
Tumor invasive depth (T1–2 versus T3–4)	2.733 (0.672–11.111)	0.16	2.805 (0.692–11.372)	0.14
Lymph node status (<1 versus ⩾1)	1.062 (0.427–2.641)	0.897	0.957 (0.382–2.401)	0.926
Distant metastasis (no versus yes)	0.192 (0.086–0.432)	<0.001^a^	0.152 (0.067–0.347)	<0.001^a^
Stage	0.145 (0.036–0.585)	0.007^a^	0.166 (0.041–0.673)	0.012^a^
Preoperative CEA (ng/ml <5 versus ⩾5)	0.768 (0.407–1.447)	0.414	0.69 (0.364–1.308)	0.256
RNF183 (high versus low)	0.384 (0.172–0.861)	0.02^a^	0.364 (0.159–0.831)	0.016^a^

Abbreviations: CEA, carcino embryonic antigen; CI, confidence interval; HR, hazard ratio; OS, overall survival; PFS, progression-free survival

a Statistically significant *P*<0.05
