# Supplementary material for: RNF183 promotes proliferation and metastasis of colorectal cancer cells via activation of NF-κB-IL-8 axis
Source: Cell Death Dis. 2017 Aug 10;8(8):e2994–. doi: 10.1038/cddis.2017.400 (PMC5596582; doi:10.1038/cddis.2017.400)
Supplement: Supplementary Information [file cddis2017400x1.docx]

**Supplementary Materials for**

**RNF183 promotes proliferation and metastasis of colorectal cancer cells via activation of NF-κB-IL8 axis**

Rong Geng, Xin Tan, Jiangxue Wu, Zhizhong Pan, Min Yi, Wei Shi, Ranyi Liu, Chen Yao, Gaoyuan Wang, Jiaxin Lin, Lin Qiu, Wenlin Huang and Shuai Chen

The supplementary materials include:

1. Two supplementary figures
2. Two supplementary tables

**Supplementary Figures**


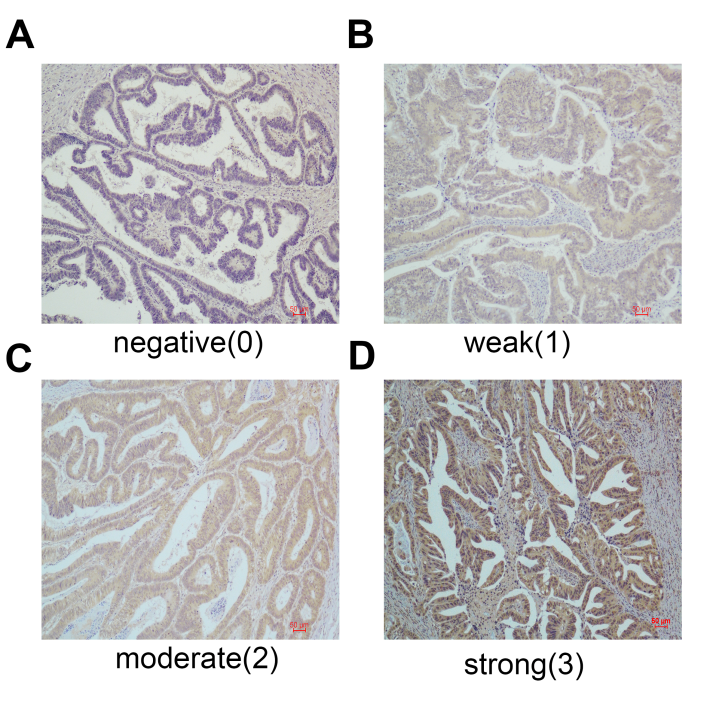


**Figure S1.** Four images represented immunoreactive score for RNF183 in 135 CRC tissue slices. The score from 0-3 indicated staining intensity containing negative staining (A), weak staining (B), moderate staining (C), and strong staining (D).

**
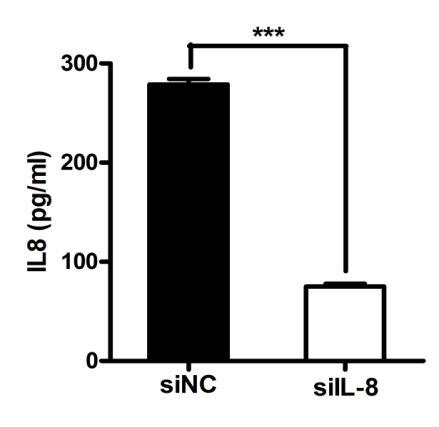
**

**Figure S2.** The level of secreted IL-8 according to transfection of siRNA targeting IL-8 in HCT116 cell. *** P<0.001.

**2. Supplementary Tables**

**Table S1.** The oligonucleotides for siRNAs against human RNF183 and IL-8.

| RNF183 siRNA-1 5'- CCACCAUGUCAUCCUGGAA-3'  RNF183 siRNA-2 5'- GCATCTTTGCCTACCTGAT-3'  IL-8 siRNA-1 5'- GCCAAGGAGUGCUAAAGAA-3'  IL-8 siRNA-2 5'- CTTAGATGTCAGTGCATAA-3' |
| --- |

| **Genes Sense primer Antisense primer** |
| --- |
| RNF183 5’-CGAAAAGCTTGAAGGACTGG-3’ 5’-TGAAGCAGCTCCAGTGAGAA-3’  IL-8 5’-CTGCGCCAACACAGAAATTAT-3’ 5’-CATCTGGCAACCCTACAACAG-3’  Snail 5’-TCGGAAGCCTAACTACAGCGA-3’ 5’-AGATGAGCATTGGCAGCGAG-3’  ZEB1 5’-AAGAAAGTGTTACAGATGCAGCTG-3’ 5’-CCCTGGTAACACTGTCTGGTC-3’  ZO1 5’-AAGTCACACTGGTGAAATCC-3’ 5’-CTCTTGCTGCCAAACTATCT-3’  E-cadherin 5’-TGAAGGTGACAGAGCCTCTGGAT-3’ 5’-TGGGTGAATTCGGGCTTGTT-3’  IL-6 5’-GTGGAGATTGTTGCCATCAACG-3’ 5’-CAGTGGATGCAGGGATGATGTTCTG-3’  iNOS 5’-CCTGGAGGTCCTGGAAGAGT-3' 5’-CCTGGGTTTCAGAAGTGGC-3'  COX2 5'-TGTCCTTTCACTGCTTTCCAT-3' 5'-TTCCATTGCTGTGTTTGAGGT-3'  IL-1β 5'-tggaccccttggtaaaagaca-3' 5'-gaagaaatcagtagagctatgaaacaaataag-3'  GAPDH 5’-CTCCTCCTGTTCGACAGTCAGC-3’ 5’-CCCAATACGACCAAATCCGTT-3’ |

**Table S2.** Primer sequence of RT-PCR
